# Status of ocular health and binocular vision among children using digital devices: a cross-sectional study

**DOI:** 10.1097/MS9.0000000000005286

**Published:** 2026-07-24

**Authors:** Nabin Pahari, Anusha Regmi, Hari Bahadur Thapa, Alisha Regmi, Mukesh Pahari, Saraswati Khadka Thapa

**Affiliations:** a Department of Intensive Care, Mercy City Hospital, Butwal, Nepal; b University of New England, Sydney, Australia; c Department of Optometry, Lumbini Eye Institute and Research Center, Bhairahawa, Nepal; d Department of Optometry, Himalyan Eye Hospital, Pokhara, Nepal; e Devdaha Medical College, Rupandehi, Nepal; f Department of Research, Lumbini Eye Institute and Research Center, Bhairahawa, Nepal

**Keywords:** children, computer vision syndrome, digital devices, dry eye, non-strabismic binocular vision anomalies

## Abstract

**Background:**

With the advancement of technologies, there is an increase in the usage of electronic devices like computers, tablets, and smartphones for both vocational and avocational purposes. This causes an increased effort for near vision, which ultimately leads to increased accommodation/convergence and decreased, infrequent blinking. Digital eye strain is a manifestation of evaporative dry eye caused by a decreased, incomplete blink rate, leading to ocular surface compromise, and asthenopic symptoms caused by problems of accommodation and convergence. This study aims to assess the status of ocular health and binocular vision among children using digital devices.

**Materials and methods:**

A hospital-based cross-sectional study was conducted among 106 children aged 9–18 years at the pediatric and strabismus department of Lumbini Eye Institute and Research Centre, Rupandehi, Nepal. All the children underwent detailed ophthalmic and binocular vision examinations. Schirmer II was performed as a quantitative test for the evaluation of dry eye. Subjects were asked about the types of digital devices they use, the amount of time they spend on them every day, how long they have been using digital devices, and the visual symptoms they experienced at the end of the day.

**Results:**

The prevalence of computer vision syndrome (CVS) was 86.8%. Headache was the most common symptom reported. CVS-related symptoms were not statistically associated with the hours of usage of a digital device (*P* = 0.092). However, the symptoms were statistically significant with age (*P* = 0.001). The prevalence of non-strabismic binocular vision anomalies was 76.4%. Our study shows vergence anomalies were higher than accommodative anomalies, with convergence insufficiency (39.6%) being the most prevalent. Dry eyes were reported in 48.1% of the subjects. Females were found to be more affected by dry eye than males (*P* = 0.013).

**Conclusion:**

This study suggests that there is a greater prevalence of CVS and convergence insufficiency in children using digital devices.

## Introduction

After the emergence of the COVID-19 pandemic and worldwide lockdown, electronic gadgets became the primary medium for online education, leading to a drastic increase in the amount of time spent on gadgets, which has caused several symptoms related to vision, the ocular surface, and the musculoskeletal system[1]. According to the American Optometric Association (AOA), “Computer vision syndrome (CVS)” is a group of eye- and vision-related problems that result from prolonged use of computers, tablets, e-readers, and cell phones[2]. As little as 2 hours of continuous digital device usage per day is enough to cause a range of eye- and vision-related problems[2].

In the world data, it is estimated that nearly 60 million people suffer from CVS[3]. Basnet *et al* showed the prevalence of CVS was 94.3% in a descriptive cross-sectional study in a tertiary care hospital of Nepal[4]. Similarly, a community-based study in Nepal shows that for children aged 3–10 years, the median screen time was 1.0 hour per day before the pandemic, which increased to 2.2 hours per day during the lockdown (*P* < 0.001). Before the pandemic, only 2.5% of children had their own screen devices; this increased to 9.8% during the outbreak[5].

Recent advances in artificial intelligence (AI) have a major influence on healthcare delivery and medical research. According to a recent machine-learning-driven global bibliometric analysis, research related to ChatGPT in medicine has expanded rapidly, with a reported monthly growth rate of 26.97% per month[6]. Similarly, deep learning models like convolutional neural networks have shown excellent diagnostic performance in analyzing ocular surface images and quantifying clinical signs associated with dry eye disease[7]. Integrating AI-driven methods into research offers a new methodological perspective for understanding the ocular health effects of digital device use among children and adolescents. Therefore, this improves accuracy and provides new opportunities for early detection and preventive strategies in pediatric eye health.

Previously, limited research has evaluated ocular health status in children pre- vs post-pandemic; however, only limited research has simultaneously assessed both subjective symptoms and objective clinical findings of CVS and binocular vision anomalies. To the best of our knowledge, no studies have been conducted in Nepal so far that have evaluated both the objective and subjective symptoms linked to increased use of digital devices in children. Therefore, the objective of this study was to evaluate the status of ocular health and binocular vision among children using digital devices.

HIGHLIGHTS

- First study in Nepal to comprehensively evaluate *both objective clinical findings* and *subjective symptoms* of computer vision syndrome (CVS), non-strabismic binocular vision anomalies (NSBVA), and dry eye in children using digital devices.
- Found an exceptionally high prevalence of CVS (86.8%) among children aged 9–18 years, with headache being the most frequently reported symptom.
- Identified convergence insufficiency as the most common binocular vision disorder, with a higher prevalence of vergence anomalies compared to accommodative anomalies.
- Documented a significantly higher prevalence of dry eye in females (*P* = 0.013), highlighting potential gender-related ocular health disparities.
- Provides critical evidence to inform public health strategies, including school-based awareness programs, screen time regulations, and the early detection of vision disorders in children.
- Strengthens the global literature by addressing a research gap in pediatric ocular health in South Asia during the post-pandemic era of increased digital device use.

The work has been reported in line with the STROCSS criteria[8].

## Methods

A descriptive cross-sectional study was conducted among 106 patients attending the Paediatric and Strabismus Department of Lumbini Eye Institute and Research Centre, Rupandehi, Nepal from May 2022 to November 2022, after obtaining ethical approval from the Institutional Review Committee (Ref: 2 502 122) of Lumbini Eye Institute and Research Centre. Informed verbal and written consent were obtained from the subjects and their guardians prior to the commencement of the study. The inclusion criteria included participants aged 9–18 years who provided consent for the study. Subjects with nystagmus, intermittent or manifest tropia, paresis or paralysis of extraocular muscles, corneal pathologies, or any anterior segment disorder were excluded from the study. Eighty-two was the estimated sample size for the study. However, our study enrolled 106 participants. A non-probability, purposive sampling method was applied.

A comprehensive proforma was developed to record the data. All the children underwent detailed ophthalmic and binocular vision examinations in the presence of their guardian. Both monocular and binocular distance visual acuity were recorded with a Snellen visual acuity chart at 6 meters, and near visual acuity was recorded with a reduced Snellen’s chart at 50 cm in a well-illuminated room. Both objective and subjective refraction were also performed. Subjects were also asked about their previous history of glasses use.

Corneal reflex was evaluated using a handheld torchlight, followed by the cover/uncover test for both distance (6 m) and near (40 cm) using the Modified Thorington test. Extra-ocular muscle function was assessed using a handheld torchlight. Stereopsis was evaluated using the Standard TNO stereo test (INSTITUTE FOR PERCEPTION, Netherlands Organization for Applied Scientific Research) at 40 cm. The Near Point of Convergence was measured with an accommodative target (letter target of the Lang fixation stick) and the penlight red/green (PLRG) procedure. The Near Point of Accommodation was assessed using the push-up method. Step fusional vergence at near (40 cm) and distance (6 m) was measured using a horizontal prism bar and a Lang letter fixation target. Negative Relative Accommodation and Positive Relative Accommodation were evaluated using loose minus and plus lenses. A lens flipper of (±2D) was used with an N8 target at 40 cm to measure monocular and binocular accommodative facility, where focusing through a minus lens and a plus lens was considered as one cycle. Monocular estimation method was performed with the subject's distance correction in place. The gradient method was used for the determination of AC/A.

Subjects were asked about the types of digital devices they use, the amount of time they spend on them every day, how long they have been using digital devices, and the visual symptoms they encountered at the end of the day, using a pre-determined questionnaire (Supplemental Digital Content File 1, available at: http://links.lww.com/MS9/B275).

The assessment of dry eye was conducted using the Schirmer II method. Values of less than 15 mm were considered indicative of dry eye[9]. The criteria for diagnosing NSBVA were based on normative values and clinical cut-off thresholds established by Hussaindeen *et al*, which include defined limits for accommodative and vergence parameters[10]. Any subject who failed more than two criteria was diagnosed with the specific anomaly. However, those who did not meet these criteria were categorized as “normal” (Supplemental Digital Content File 2, available at: http://links.lww.com/MS9/B276).

All the data were entered into Microsoft Excel, and statistical analysis was carried out using the Statistical Package for Social Sciences (SPSS) software, version 20.0. Descriptive statistical measures, such as median and interquartile range for continuous variables, and frequency and percentage for categorical variables, were computed. Appropriate dummy tables, pie-charts, bar diagrams, and histograms were used during the analysis. The association between categorical variables was analyzed using the Chi-square test. *P* value was calculated under the predetermined level of significance (< 0.05, significant).

## Results

A total of 106 subjects were enrolled in the study, who attended a Paediatric and Strabismus department for a general eye examination during the study period. The median age of the included patients was 16 years. Among them, 27 (25.5%) were male patients and 79 (74.5%) were female patients. Among 212 eyes of 106 participants, 72.6% were emmetropic, 15.2% were astigmatic, 9.4% were myopic, and only 2.8% were hyperopic. Our study has shown that the frequency of participants who had a history of wearing spectacles is 18 (17%), and 88 (83%) of the participants did not have a history of wearing spectacles.

The most commonly used digital device among users was mobile phones, with 59 participants (55.7%) using them, and 41 participants (38.7%) reported using more than one device. Out of all participants, the maximum number (37.7%) had been using digital devices for 1–2 years, followed by 33% for 3–4 years, and 26.4% for more than 4 years. Similarly, most participants (31.1%) were using digital devices for 4–5 hours per day, while 15.1% were using them for more than 7 hours per day.

**Figure 1.**
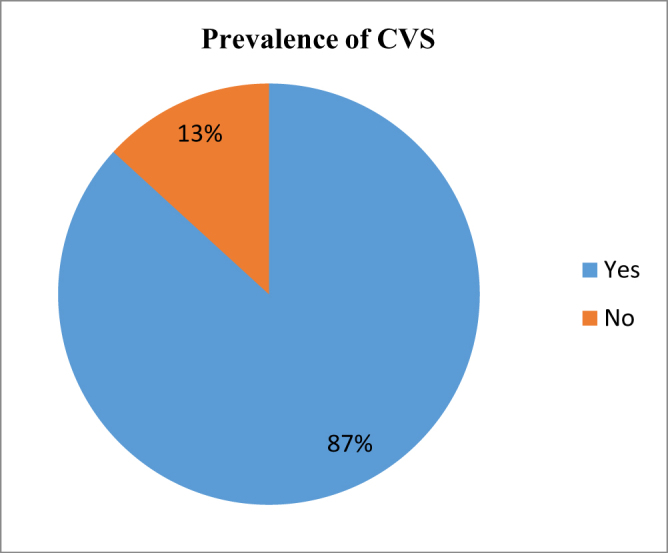
Distribution of participants according to CVS.

Figure 1 shows that the prevalence of computer vision syndrome (CVS) was 86.8%. CVS-related symptoms were not statistically significant with the hours of usage of a digital device (*P* = 0.092).

Table 1 shows that the prevalence of NSBVA was 76.4%, while 23.6% fell under the category of normal.

**Table 1:** Distribution of participants according to status of binocular vision.

Diagnosis	*N* (%)
Within normal limit	25 (23.6%)
NSBVA	81 (76.4%)

**Figure 2.**
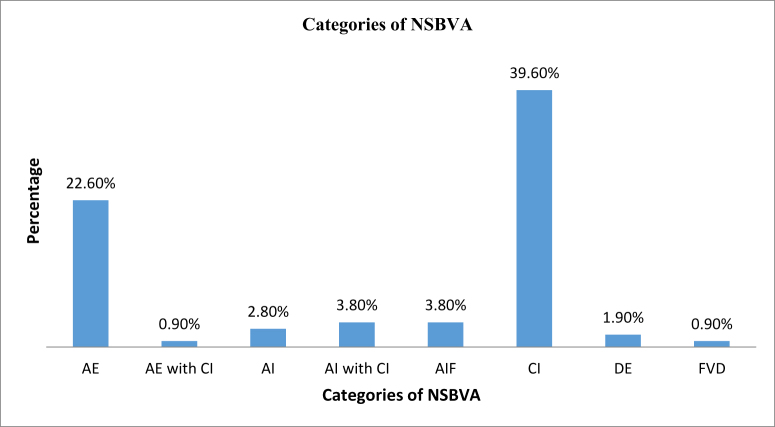
Distribution of participants according to the category of NSBVA.

Out of all the subjects with NSBVA, 45 (42.45%) participants presented with vergence anomalies, 31 (29.24%) participants with accommodation anomalies, and 5 (4.71%) participants with both vergence and accommodation anomalies. Figure 2 shows that the most common dysfunction of NSBVA was convergence insufficiency (39.6%), followed by accommodative excess (22.60%).

**Figure 3.**
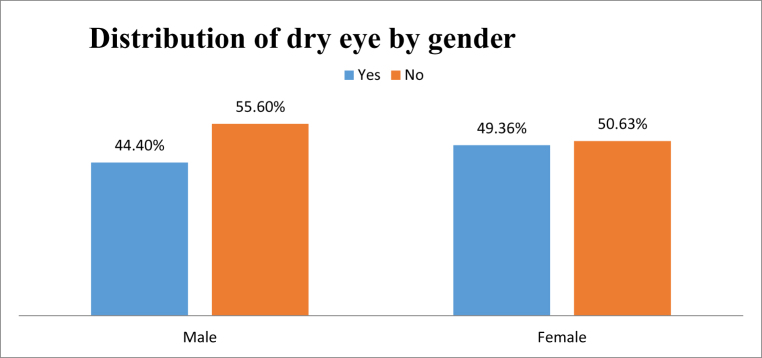
Distribution of dry eye according to gender.

Table 2 shows that only 48.1% of subjects had dry eye. Figure 3 indicates that females were found to be more affected by dry eye than males.

**Table 2:** Distribution of participants according to dry eye.

Dry eye	*N* (%)
Yes	51 (48.1%)
No	55 (51.9%)

**Figure 4.**
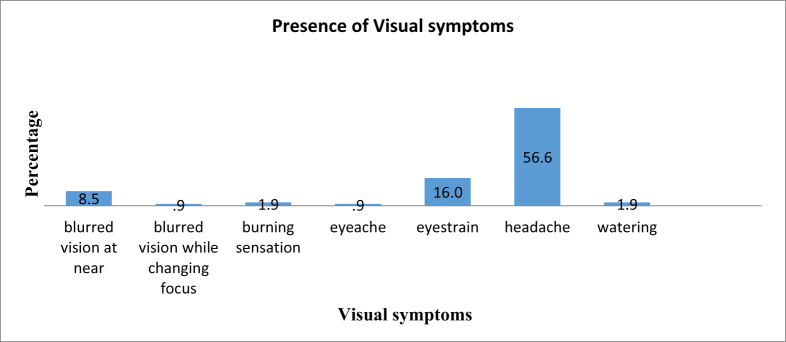
Distribution of subjects with the presence of visual symptoms.

Different categories of visual symptoms were reported in the study. Most subjects reported one or more visual symptoms. Table 3 shows that out of 106 subjects, 92 (86.8%) complained of having visual symptoms, while 14 (13.2%) subjects didn’t have any symptoms. A maximum of 60 participants (56.6%) complained of having headaches, while 17 (16%) reported eyestrain. Figure 4 shows different categories of visual symptoms, along with the percentage of complaints reported by the subjects. Furthermore, Table 4 shows that the CVS symptoms were statistically significant with age [likelihood ratio (2, *N* = 106) = 13.526, *P* = 0.001].

**Table 3:** Distribution of response about visual symptoms.

Symptoms	Frequency	Percentage
Yes	92	86.8%
No	14	13.2%
Total	106	100%

**Table 4:** Association between CVS symptoms and age of participants.

Chi-square tests	Value	df	Asymp. Sig. (2-sided)
Pearson Chi-square	9.313^a^	2	.009
Likelihood ratio	13.526	2	.001
Linear-by-linear association	5.421	1	.020
*N* of valid cases	106		

^a^ Two cells (33.3%) have an expected count of less than 5. The minimum expected count is 1.32.

## Discussion

The goal of this study was to determine the prevalence of CVS in children who were using digital devices. This study has associated visual symptoms and findings of the binocular vision status of children using digital devices, along with the diagnostic examination of dry eye.

A notably high prevalence of CVS was observed in this hospital-based study, which aligns with other studies. In some ways, the findings mirrored those of studies by Poudel S. *et al* (82.5%) and Reddy S.C. *et al* (89.9%)^[^11,12^].^ Similar findings were determined by Basnet *et al*. in their study, which found that 300 (94.3%) people had one or more digital eye strain (DES) symptoms[4]. This may be because adults and college students engage in digital work at higher rates than children. In addition, according to Iqbal *et al*, 86% of the medical students had one or more symptoms of CVS[3].

Our study found that CVS-related symptoms were not statistically significant with the number of hours spent using a digital device (*P* = 0.092), in contrast to Wadhwani *et al*’s study (*r* = 0.15; *P* = 0.036), which found a significant association between the increase in the number of activities and the onset of CVS symptoms in children[13]. Since no objective tests were performed in Wadhwani’s online study and the participants were parents of children, this could lead to information biases. Similarly, Seresirikachorn *et al* discovered that the total hours of digital device usage were correlated with the severity of CVS (*P* < 0.001)[14]. However, Seresirikachorn conducted a cross-sectional, online, questionnaire-based study with 2476 students, a larger sample size than our study. These differences may be influenced by the relatively small sample size, self-reported responses from participants, and children’s adaptive nature to visual discomfort in the present study.

However, our study showed that CVS-related symptoms were significantly associated with age (*P* = 0.001), which was similar to the study by Abuallut *et al* (*P* < 0.05)[15]. Likewise, Rahman *et al* showed that being less than 27 years old was a significant predictor for CVS[16]. Therefore, the findings of the current study are consistent with the results of existing studies, indicating that younger populations may experience a higher burden of CVS-related symptoms.

Our study showed a considerable proportion of subjects (48.1%) had dry eyes. Comparable prevalence rates have been reported in previous studies, including Tawil *et al*, who concluded the prevalence of dry eye was 51.5% (mild, moderate, and severe)[17]. Similarly, Mufti *et al* concluded the prevalence of dry eye is 55.6%[18]. A slightly higher incidence of dry eye in some studies might be attributed to methodological differences, as the Mufti *et al* study used Schirmer I as a diagnostic test, which may lead to higher detection rates due to reflex tearing.

In the present study, dry eye symptoms were more commonly observed in females than males (*P* = 0.013), which was similar to the study by Bahkir *et al*, which also concluded that females were more affected than males (*P* = 0.009)[1]. Wang *et al* also showed that the incidence rate of dry eye and immune-related dry eye in women was significantly higher than that in men[19]. This might be due to autoimmune and hormonal factors that have been linked with ocular surface disorders^[^19,20^].^ These findings might also be due to uneven gender distribution, with a higher proportion of females in our study.

In our study, the majority of the subjects were under the category of NSBVA. Previous studies by Scheiman *et al* have similarly reported that NSBVA is almost 8.5–9.7 times more common than the prevalence of ocular disease in children between 6 and 18 years of age[21]. Hussaindeen *et al* also showed a potential age effect, with the prevalence of non-strabismic anomalies of binocular vision increasing from 25.1% in the 7–12 years age group to 36.2% in the 13–17 years age group[22]. This may be related to the increased near-visual demands experienced by older children during academic activities.

Among the 105 participants with NSBVA in this study, 45 (42.45%) participants presented with vergence anomalies, 31 (29.24%) participants with accommodation anomalies, and five (4.71%) participants with both vergence and accommodation anomalies. Similar patterns were observed in a study by Shin *et al*, which showed 9% of participants with accommodative dysfunctions, 13.2% with vergence dysfunctions, and 6.3% with combined accommodative and vergence dysfunctions among 9–13-year-old children[23]. However, numerous studies show that accommodative anomalies are more common than vergence dysfunctions ^[^23–25^]^ Our study highlights the predominance of vergence anomalies, with convergence insufficiency being the most common, which may be attributed to the increased near-visual demands experienced by school-aged children during prolonged reading and digital device use. These findings are supported by numerous studies^[^26–28^]^.

The integration of AI technologies into pediatric eye health research may, therefore, provide several advantages for future studies. Large language models, such as ChatGPT, have gained increasing attention for their ability to identify clinical signs of ocular surface disease, indicating that AI-driven approaches may present new opportunities for the objective evaluation and monitoring of ocular conditions like dry eye and DES in large populations.

### Strengths and limitations

This study is the first study so far that will contribute to providing insight into both objective and subjective symptoms linked to increased use of digital devices. Moreover, the results demonstrate that there is a greater prevalence of CVS among children, suggesting the greater impact of digital devices on ocular health.

However, this might have been caused by pre-existing ocular issues in children. The major limitation of the study was that the association between hours of usage of digital gadgets and the increasing prevalence of CVS couldn’t be established due to the small sample size, and the link between outcome and exposure could not be determined because both were examined at the same time. This is a hospital-based study with a non-probability purposive sampling technique, which may have introduced selection bias and limits the generalizability of the findings to the wider pediatric population in Nepal. Additionally, the absence of a control group limits the ability to compare findings with children who have minimal or no exposure to digital devices. To gain a better understanding of the link between exposure to digital devices and pediatric ocular health, future large-scale, community-based longitudinal studies incorporating the use of objective screen-time monitoring tools are essential.

## Conclusion

This study suggests a greater prevalence of CVS, dry eye, and NSBVAs among children aged 9–18 years attending a tertiary eye care center in Nepal. CVS-related symptoms were statistically significant with age, suggesting a higher frequency of CVS-related symptoms in children of this age group. However, variations in the findings may be due to the cross-sectional design, sampling methods, and lack of adjustment for potential confounding factors; the findings indicate association rather than causation. If identified early, these anomalies can be effectively managed with prism prescription, optical correction, and vision therapy. Hence, awareness campaigns are essential to educate students about the long-term impacts of digital screens on ocular health, and healthy digital device practices following the 20/20/20 rule – every 20 minutes looking 20 feet away from the screen and blinking eyes for at least 20 seconds – should also be promoted.

## Data Availability

Data are available upon reasonable request.

## Research registration unique identifying number (UIN)

ResearchRegistry11467
